# Analytical Validation of a DNA Methylation Biomarker Test for the Diagnosis of Barrett’s Esophagus and Esophageal Adenocarcinoma from Samples Collected Using EsoCheck^®^, a Non-Endoscopic Esophageal Cell Collection Device

**DOI:** 10.3390/diagnostics14161784

**Published:** 2024-08-15

**Authors:** Abhisek Ghosal, Suman Verma, Ivy T. Le, Victoria T. Lee, Brian J. deGuzman, Lishan Aklog

**Affiliations:** 1 Lucid Diagnostics, 360 Madison Ave., Floor 25, New York, NY 10017, USA; abg@luciddx.com (A.G.); ile@luciddx.com (I.T.L.); vtl@pavmed.com (V.T.L.); bjd@pavmed.com (B.J.d.); la@pavmed.com (L.A.); 2 Lucid Dx Labs, 14 Orchard Road, Lake Forest, CA 92831, USA; 3 PAVmed Inc., 360 Madison Ave., Floor 25, New York, NY 10017, USA

**Keywords:** analytical validation, Barrett’s esophagus (BE), College of American Pathologists (CAP), Clinical Laboratory Improvement Amendments (CLIA), cyclin A1 (*CCNA1*), vimentin (*VIM*), DNA methylation biomarker, early detection, esophageal adenocarcinoma (EAC), laboratory-developed test (LDT)

## Abstract

Barrett’s esophagus (BE) is a known precursor to esophageal adenocarcinoma (EAC). Guidelines recommend BE screening in populations with multiple risk factors, for which non-endoscopic esophageal cell collection with biomarker testing is considered as an acceptable alternative to esophagogastroduodenoscopy (EGD). The aim of this study was to evaluate analytical performance characteristics of EsoGuard^®^ (EG), a DNA methylation biomarker assay, as a laboratory-developed test (LDT) in esophageal samples collected with the swallowable EsoCheck^®^ (EC) device. EG is a next-generation sequencing (NGS) assay that evaluates methylated *vimentin* (*VIM*) and *cyclin A1* (*CCNA1*), clinically validated biomarkers for the detection of BE and EAC. The studies were conducted according to standards of College of American Pathology (CAP), Clinical Laboratory Improvement Amendments (CLIA), and New York (NY) state requirements for the analytical validation of molecular assays. Comparison to Sanger sequencing showed that EG was 100% accurate at all 31 CpG sites evaluated by the assay. The analytical sensitivity, specificity, and accuracy of the assay were 89%, 100%, and 96%, respectively. Intra- and inter-assay precision was 100%. The limit of detection (LOD) was 1 in 400 methylated cells, and the reference range was 84%. In summary, EsoGuard demonstrates high analytical accuracy, repeatability, and reproducibility in samples collected using the EsoCheck device.

## 1. Introduction

Esophageal adenocarcinoma (EAC) is the second most lethal cancer in the United States (U.S), with an over 500% increase in the last four decades and a dismal average 5-year survival of only 20% [1,2,3,4]. Barrett’s esophagus (BE) is the only known direct precursor to EAC [5,6]. In contrast to EAC, BE can be successfully treated using endoscopic techniques, with 80–90% success rates [7,8,9]. Screening for BE is supported by multiple gastroenterology society guidelines and clinical practice recommendations; however, the penetrance of endoscopic screening has traditionally been poor [10,11,12,13]. To improve the compliance and accessibility of BE screening, the American College of Gastroenterology (ACG) and American Gastroenterology Association (AGA) now consider non-endoscopic esophageal cell collection in combination with biomarker testing as an acceptable alternative to screening endoscopies [14,15].

EsoGuard^®^ (EG) is a next-generation sequencing (NGS) DNA methylation biomarker assay that investigates methylation signatures in the *VIM* and *CCNA1* genes and is performed in a College of American Pathology (CAP)-accredited, Clinical Laboratory Improvement Amendments (CLIA)-certified, and New York state (NYS)-licensed laboratory (LucidDx Labs, Lake Forest, CA, USA). It is the only commercially available biomarker assay available in the U.S for the non-endoscopic detection of BE and EAC. 

EG investigates the specific methylation signature of 31 CpG sites on the *VIM* and *CCNA1* genes, which have been shown as promising biomarkers for of all stages of BE as well as EAC [16]. The performance of EG’s combined *VIM* and *CCNA1* two-gene methylated biomarker panel for the detection of BE/EAC was first published in 2018 [16]. In this study, the EG panel was performed in an academic laboratory and demonstrated a clinical sensitivity of 88% and clinical specificity of 92% in samples collected with the non-endoscopic EsoCheck^®^ (EC) balloon device (*n* = 86) (Table 1). More recently, three additional independent clinical validation studies (one case–control and two single-arm studies in the intended use population) reported clinical performance of the commercial EG assay, where cell samples were collected using EC and then preserved and transported at ambient temperature to LucidDx Labs [17,18,19]. The clinical performance of EG for detecting BE/EAC in these recent studies was highly consistent with the 2018 study (Table 1). 

The EG assay reports qualitative, binary results based on a clinically validated cut-off of >1% methylation for *VIM* or >0.5% methylation for *CCNA1* [16,18]. A positive EG result suggests the presence of intestinal metaplasia or disease along the BE progressive spectrum (non-dysplastic BE, dysplastic BE, and EAC), and therefore should be further investigated and staged with EGD. In clinical validation studies, EG has demonstrated a high negative predictive value (NPV, 98.6%), suggesting that a negative result likely does not warrant further diagnostic evaluation [17,19]. Clinically, the assay has been used as a triage to EGD when evaluating patients who meet guideline criteria for BE screening [20,21,22]. In a recently published real-world registry data, binary EG results were reported in 94% of samples, with a positivity rate of 14.1% [22].

Samples for EG analysis are collected non-endoscopically with EsoCheck (EC), an FDA 510(k)-cleared, swallowable, balloon capsule device that circumferentially collects surface cells from a targeted region of the esophagus. It is performed without sedation and allows for rapid, in-office collection. Cell samples collected with EC can also be utilized for cytology or other diagnostic testing. Published real-world experience with EC shows a cell collection success rate of 96.9–99.6% [20,21,22]. 

The aim of this manuscript is to summarize the analytical performance characteristics of EG, which has been validated as a laboratory-developed test (LDT) per the standards of CAP, CLIA, and the NY state. This includes results of studies conducted to determine analytical accuracy, analytical sensitivity, analytical specificity, linearity, lower limit of detection (LLOD), limit of blank (LOB), inter and intra-assay precision, reference intervals, and the reportable range for the EG assay. Additionally, we present data for sample stability in preservative media during room temperature transportation and interfering substance studies.

## 2. Materials and Methods

### 2.1. Cell Culture

The NCI-H1975 cell line (CRL-5908; ATCC, Manassas, VA, USA) and SK-TG-4 cell line (11012007; Millipore Sigma, Burlington, MA, USA) were utilized to create contrived specimens. The cells were cultured in an RPMI-1640 medium (30-2001; ATCC, Manassas, VA, USA) supplemented with 10% fetal bovine serum (30-2020; ATCC, Manassas, VA, USA) in the presence of a 1× antibiotics mixture (penicillin and streptomycin). The cells were passaged either in a 1:2 or 1:3 ratio following trypsinization, and the total cell count was determined by trypan blue staining with counting in the Countess 3 FL-Automated Cell counter (Thermo Fisher Scientific Inc., Waltham, MA, USA). Each of the cell lines (SK-TG-4 (100% methylated) and NCI-H1975 (0% methylated)) were mixed in a 1:100 ratio for creating a 1% spike-in. 

### 2.2. Sample Collection

Esophageal cell samples were collected using EC as per the device instructions for use (IFU). Each sample was placed in a proprietary liquid preservative, and the cells were suspended via shaking (or mixing) and then transported and stored at room temperature until the time of DNA extraction.

### 2.3. DNA Extraction from EsoCheck Balloon Samples

Cells from EC-collected samples were harvested from the preservative media by centrifugation at 4000 rpm for 5 min after removing the EC balloon. Cells were lysed at 56 ± 2 °C in a shaking heat block (1200 rpm for 30 min) in the presence of proteinase K. The lysed cells were subjected to automated bead-based purification using the NuCleoMag Tissue Kit (Macherey-Nagel, Dueren, Germany) and KingFisher Apex instrument (Thermo Fisher Scientific Inc., Waltham, MA, USA). Eluted DNA was quantified by the Qubit™ dsDNA HS Assay reagent (Thermo Fisher Scientific Inc., Waltham, MA, USA) following a standard protocol. 

### 2.4. EsoGuard Assay

The extracted DNA was bisulfite converted using the EZ DNA methylation lightning kit (Zymo Research, Irvine, CA, USA) and cleaned using the KingFisher Apex instrument (Thermo Fisher Scientific Inc., Waltham, MA, USA). Purified bisulfite-converted DNA was tested in either singleplex or multiplex for the *VIM* and *CCNA1* genes using primers and polymerase chain reaction (PCR) conditions as previously described [16]. In multiplex testing, each bisulfite-converted DNA sample was divided into three technical replicates prior to PCR. The indexed amplicon library was pooled from multiple samples and subjected to AMPureXP (Beckman Coulter Life Sciences, Brea, CA, USA) bead clean up, followed by end-repair, an A-tailing step using the NEBNext^®^ Ultra™ II kit (New England Biolabs, Ipswich, MA, USA), and dual index adapter ligation using NEXTflex^®^ Dual-Indexed DNA Barcodes (Perkin Elmer Inc., Waltham, MA, USA). The final library was quantified by the Qubit™ dsDNA HS Assay (Thermo Fisher Scientific Inc., Waltham, MA, USA) and sequenced on the MiSeq™ or NextSeq 1000 sequencing platform (Illumina Inc., San Diego, CA, USA). 

Each EG assay run included one low positive control (1% contrived cells), one negative control (0% contrived cells), one PCR blank, and one bisulfite blank. Each run was evaluated for controls prior to analyzing sample results. 

### 2.5. Bioinformatics Analysis

DNA sequencing reads were processed in the data analysis pipeline as described in a previous publication [16]. The methylation status of each gene was determined based on cut-offs established from early clinical validation studies [16,18]. The final EG result was considered positive if methylation on either or both the *VIM* or *CCNA1* genes met the cutoff for positivity, and negative if both genes failed to meet the cutoff. In the case of triplicate testing, the methylation status of each sample was determined based on a majority call from three technical replicates of the sample. As quality control (QC) parameters, each sample was evaluated for mapping efficiency, bisulfite conversion efficiency, percent reads covering the full-length CpG, and depth of coverage of each gene (>10,000× coverage). If a sample failed any of these QC criteria, the sample was reported by the pipeline as non-diagnostic.

### 2.6. Sanger Sequencing

The extracted DNA from SK-TG-4 and NCI-H1975 were bisulfite-converted as described above and amplified with specific primers for *VIM* and *CCNA1* separately. The amplified PCR products were cleaned by the NucleoSpin Gel and PCR cleanup kits (Macherey-Nagel, Dueren, Germany) following the manufacturer’s recommendations, and the cleaned PCR product was sent to an outside sequencing laboratory (Genewiz from Azenta Life Sciences Inc., South Plainfield, NJ, USA) for sequencing of both forward and reverse strands. The full-length DNA sequence was aligned by a basic alignment tool (blastn, NCBI, Bethesda, MD, USA). 

### 2.7. Statistical Methods

The sample mean was calculated by averaging the replicates and plotted with a standard deviation. The % coefficient of variation (%CV) was calculated by dividing the standard deviation with the sample mean. Assay linearity was assessed using the coefficient of determination (R^2^) value from a scatterplot.

## 3. Results

### 3.1. Accuracy of Methylation Calling

The aim of this study was to evaluate the accuracy of the sequencing calls made at all 31 CpG sites (10 *VIM* CpG sites and 21 *CCNA1* CpG sites) investigated by the EG assay. The accuracy was assessed by a comparison to Sanger sequencing as the “gold standard”. A total of 62 unique data points were evaluated (two cell lines at 31 CpG sites across two gene loci) in triplicates. All 31 CpG sites showed methylation levels as expected based on Sanger sequencing (Table 2A,B, Supplementary Figure S1). The methylation percentages at each CpG site for both the *VIM* and *CCNA1* genes were within the expected range of variance, considering that Sanger sequencing is only semi-quantitative, with a limit of resolution of approximately 15–30% allele fractions. The EG analysis pipeline provides methylation % based on each sequencing read, evaluating all 10 CpG sites for *VIM* and 21 CpG sites for *CCNA1*. When data from this pipeline were analyzed, they were highly concordant with Sanger sequencing across both the *VIM* and *CCNA1* regions (Table 2C).

### 3.2. Analytical Sensitivity, Specificity, and Accuracy

To evaluate the analytical accuracy, analytical sensitivity, and analytical specificity of the assay, studies were performed in two phases. In phase 1, the singleplex EG assay (i.e., *VIM* and *CCNA1* genes amplified individually) was validated using an outside reference lab as the comparator while, in phase 2, the validated singleplex assay was used as the comparator for the multiplex assay. The multiplex assay refers to the assay after a process update allowing the *VIM* and *CCNA1* genes to be amplified in a single tube rather than separately (no modifications to the primers, methylation cutoffs, or the assay algorithm). In phase 1, samples were exchanged with Case Western Reserve University (CWRU), where the assay was originally developed. The accuracy dataset consisted of blinded patient DNA specimens collected using EC (*n* = 49) and contrived cell line mixtures (*n* = 40). The overall analytical sensitivity, specificity, and accuracy of the EG assay were 99%, 87%, and 96% when compared to the CWRU reference lab (Table 3A–C). The balloon samples showed an analytical sensitivity, specificity, and accuracy of 97%, 81%, and 92%, respectively, while the contrived cell lines showed 100% analytical sensitivity, specificity, and accuracy (Table 3C). 

In phase 2, the EG assay was upgraded to allow for the multiplex testing of the *VIM* and *CCNA1* genes and sequencing on the MiSeq™ or NextSeq 1000 platforms based on sample volume. Additionally, samples were tested in triplicates instead of singly. The testing of the analytical sensitivity, specificity, and accuracy of the multiplex EG assay was performed by comparing against the singleplex assay in a total of 77 EC-collected samples (27 positive and 50 negative samples according to the EG singleplex assay). The multiplex assay displayed 89% analytical sensitivity, 100% analytical specificity, and 96% analytical accuracy (Table 4). Three false negative samples were discordant for the *VIM* gene only, where methylation percentages were close to the cutoff of 1.0% (1.1%, 1.1%, and 1.2%; Supplementary Table S1).

### 3.3. Bioinformatic Pipeline Accuracy

The aim of this study was to evaluate the accuracy of the EG bioinformatics pipeline. It was evaluated against the reference laboratory (CWRU) bioinformatics pipeline by testing the same data set (*n* = 43) between the two sites. The accuracy was 100% for the pipeline, with an R^2^ of 1 suggesting that identical data were obtained using both pipelines. Recent updates to the pipeline at LucidDx Labs, including updates to the bioinformatics tools and automated initiation of data analysis, were all validated against the previous version of the pipeline and showed an accuracy of 100% (*n* = 207) (Supplementary Figure S2).

### 3.4. Accuracy for Sequencing Platform

To increase scalability, the EG assay was validated on the NextSeq 1000 sequencing platform which has a higher throughput per run than MiSeq on which the initial assay was developed. The accuracy of the NextSeq 1000 sequencing platform was compared against the MiSeq platform by testing the same library on both instruments. The sample cohort consisted of 62 EC-collected samples (33 positive and 29 negatives per the MiSeq platform) and 30 contrived cell line samples. The accuracy when comparing data derived from the platforms was 100% with an R^2^ of 0.998 for *VIM* and 0.998 for *CCNA1* (Supplementary Figure S3). 

## 4. Intra-Assay and Inter-Assay Precision

### 4.1. EsoGuard^®^ Assay Precision

Precision studies were performed to evaluate the robustness of results obtained by the EG assay within each run (intra-assay) and between runs (inter-assay). The intra-assay and inter-assay precision were determined by testing a sample cohort of 10 unique samples (four contrived and six EC-collected samples). The contrived sample set consisted of a mixture of positive and negative cell lines mimicking medium- and low-positive samples, and included one negative sample (10%, 1%, 0.5%, and 0%). The EC samples consisted of three positive and three negative clinical discard specimens. The intra-assay precision was determined by testing each sample a minimum of three times on the same day, in the same run, and by a single operator. Inter-assay precision was determined by testing all ten samples at least three times, on three different runs, on three different days, by different operators. The inter and intra-assay precision for the EG assay were 100% for all samples; the %CV ranged from 1 to 34% for *VIM* and 2 to 67% for *CCNA1* in positive samples and was 0% for all negative samples (Table 5 and Table 6). 

### 4.2. Assay Linearity and Limit of Detection (LOD)

**Figure 1:**
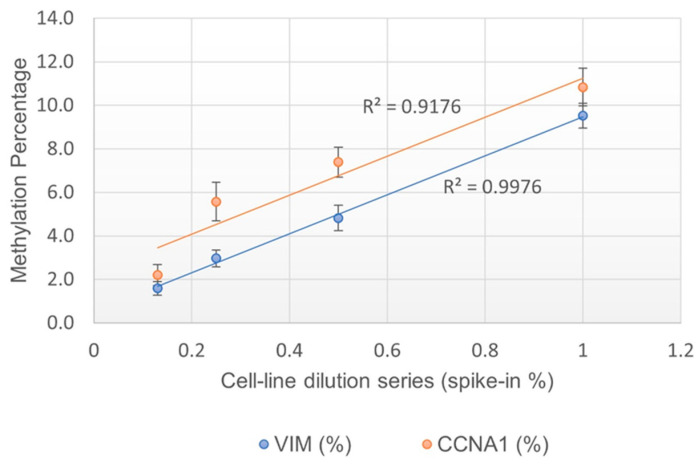
Linearity of methylation% obtained for *VIM* and *CCNA1* genes.

The purpose of this study was to assess if the methylation % obtained by the EG assay was linearly correlated with the percentage of methylated cells present in the sample. The assay linearity was measured by testing serially diluted contrived cell line mixtures (1%, 0.5%, 0.25%, and 0.13% spike-in). Each dilution was tested in at least 20 replicates. A positive correlation was observed for both the *VIM* and *CCNA1* genes, with an R^2^ at 0.998 and 0.918, respectively, suggesting that the assay is indeed linear (Figure 1). The LOD of the assay was determined to be at 0.25% spike-in, or 1 methylated cell in the background of 400 unmethylated cells, since 0.25% was the lowest dilution at which >95% of data points were positive (Table 7).

### 4.3. Assay Input Range

The purpose of this study was to establish the range of sample DNA input amounts that can provide valid results in the EG assay. The study was performed using EC-collected clinical samples (three EG-positive and three EG-negative) along with contrived specimens mimicking low-positive samples (0.5% and 1%, spike-in). All samples were tested at 50, 80, 100, and 300 ng of DNA input. EG concordance was 100% for the full range. However, for the commercial EG assay, the minimum DNA requirement is set at 100 ng to remain consistent with published clinical validation studies that were performed at this input limit. The maximum DNA input range has been established at 300 ng.

### 4.4. Reference Range

The purpose of this study was to establish expected EG results in a patient population that does not have BE or EAC. The assay reference range was determined by testing 82 samples that were confirmed as clinically negative for BE/EAC by EGD. In the sample cohort, 69 samples were detected by EG as true negatives, whereas 13 samples were false positives by the EG assay. As such, the EG assay specificity was calculated to be 84.1% in these non-diseased patients (Table 8).

### 4.5. Reportable Range:

The reportable range is the range of expected results for a diagnostic test. EG reports a qualitative, binary (“Positive” or “Negative”) result. Infrequently, cell samples may provide an insufficient amount of DNA for EG analysis, or the samples may have quality issues prohibiting analysis (occurring in 3–6%) [19,20,21]. These results are reported as “Quantity Not Sufficient” (QNS) or “Unevaluable”, respectively. If this occurs, patients and providers may repeat the EC cell collection to provide an analyzable sample for EG.

### 4.6. Limit of Blank (LOB)

Limit of blank studies were performed to establish the highest methylation % that can be obtained by EG in samples that are known to not have methylation at the 31 CpG sites investigated by the assay. Negative cell line controls with known methylation status (H-1975) were tested in multiple replicates across multiple runs (*n* = 60) by different operators. Methylation percentages for all the data points were reported at 0% for both *VIM* and *CCNA1*

### 4.7. Sample Stability in Preservative Media

**Figure 2:**
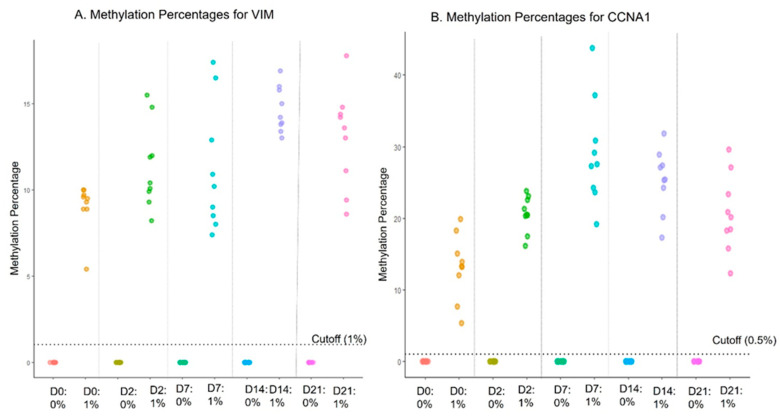
Sample stability in transport media at room temperature.

The purpose of this study was to establish the length of time that the methylation signal is stable in samples after collection and during transport to provide reliable EG results. Sample stability in the proprietary preservative was tested for up to 21 days of storage. Low-positive cell line spike-ins (1% contrived specimens) were used along with negative cell lines (0%). Each cell line sample was incubated for 0, 2, 7, 14, and 21 days (*n* = 6 for each) in preservative media at room temperature (25 ± 3 °C). Data from the EG assay showed 100% concordance to the expected results for both positive and negative samples for all time points up to 21 days (Supplementary Table S2). The dot plot shows the distribution of methylation reads on different days for both genes (Figure 2). Further, the potential effect of any extreme temperatures during shipping was evaluated by incubating the low-positive and negative cell line controls at −20 °C, 4 °C, room temperature (25 ± 3 °C), 37 °C, and 50 °C, for 2 days followed by incubation at room temperature for an additional 12 days. The assay displayed 100% concordance at all temperatures (Supplementary Table S3).

### 4.8. Interference Testing

**Figure 3:**
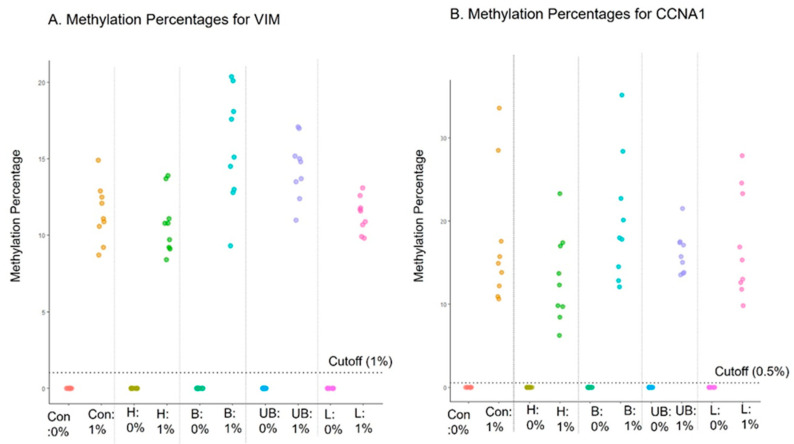
EsoGuard assay performance in the presence of interfering substances. Con, H, B, UB, and L represent control, heme, conjugated bilirubin, unconjugated bilirubin, and triglyceride-rich lipoproteins, respectively.

The purpose of interference testing is to evaluate if assay results can be impacted by any external substances that may be present in the samples. Interference with the EG assay by hemolysate (heme), bile (conjugated or unconjugated bilirubin), and triglyceride-rich lipoprotein was evaluated, as these substances are generally present in blood and the proximal gastrointestinal tract. In case large amounts of blood or these other compounds are ever present in a sample collected with EC, it would be important to discern the potential impact on the accuracy of EG results. Known negative (0% methylation; *n* = 3) and low-positive contrived cell line samples (1% methylation; *n* = 3) were incubated in the preservative media for 2 days with the interfering substances and tested with the EG assay after DNA extraction. The methylation data from the EG assay showed 100% concordance for both negative and positive samples, indicating that no interference was observed from any of the tested substances (Figure 3).

### 4.9. Percentage of Samples near Cutoff

The purpose of this study was to evaluate if binary test results are appropriate for samples with a methylation % near the cutoff, or whether an “indeterminate” result should be assigned to these patients. As with any qualitative, binary assay, EG’s precision may be lower near the cutoff (as suggested by a higher %CV closer to the cutoff). We quantified the percentage of samples near the cutoff, i.e., the methylation “grey zone”, in a real-world cohort to assess whether a binary assay was justified or whether an “indeterminate” result should be reported for samples falling in this range. First, the grey zone for each gene was calculated using EC specimens with known EGD results (8 EGD positive for BE and 82 EGD negative for BE). Positive samples were supplemented with contrived low-positive specimens (*n* = 21) near the cutoff. A total of 333 EG data points were evaluated (87 positive and 246 negatives). The grey zone for *VIM* was determined to be 0.58–1.95% and, for *CCNA1*, the gray zone was 0.24–1.57% (95%CI). A total of 2617 real-world samples (495 EG-positive and 2122 EG-negative) that were previously tested in the clinical lab were retrospectively evaluated to determine whether the methylation percentage fell within the grey zone for either *VIM, CCNA1,* or both. The results showed that 12.2% of samples were in the grey zone for *VIM* and 1.3% for *CCNA1*. Only 1.7% of samples were in the grey zone for both *VIM* and *CCNA1*. The accuracy of EG results in the grey zone was further assessed by comparing EG binary results with EGD results in a separate cohort of samples with known endoscopy results. In a sample set of 93 evaluable samples tested as part of a clinical study conducted by Lucid Diagnostics, 2 samples (2.1%) were in the grey zone for both *VIM* and *CCNA1*, 25 (27%) were in the grey zone for *VIM* alone, and 3 (3.2%) were in the grey zone for *CCNA1* alone. When compared against EGD results, 57% of these grey zone samples had accurate EG results (10% true positive and 47% true negative), 40% were false positive, and 3% were false negative (data on file; Lucid Diagnostics Inc., New York, NY, USA). This demonstrates that the assay cutoff is optimized for sensitivity over specificity.

## 5. Discussion

EsoGuard^®^ is a novel biomarker assay that investigates methylation at 31 CpG sites in the regulatory regions of the *VIM* and *CCNA1* genes, which have been correlated with metaplastic and neoplastic changes in esophageal cells [16]. It utilizes a proprietary algorithm that calculates the degree of methylation per read for both genes and provides the percentage of methylated sequencing reads. If either *VIM* or *CCNA1* or both genes demonstrate methylated reads above a pre-established cutoff, the EG assay result is reported as positive [18]. The assay’s performance in detecting BE and EAC in esophageal cell samples collected non-endoscopically using the EsoCheck^®^ device ( Lucid Diagnostics Inc., New York, NY, USA) has been clinically validated in four separate studies utilizing EGD with biopsies as the gold-standard diagnostic comparator [16,17,18,19]. A positive EG result suggests that the presence of cellular changes is associated with BE or EAC and should be followed with confirmatory EGD for a definitive diagnosis and histopathologic definition of the underlying disease. A negative result suggests that BE or EAC is not present [20,22]. The assay does not distinguish different stages of disease along the Barrett’s metaplasia-to-neoplasia spectrum. 

The aim of this study was to evaluate the analytical performance characteristics of the EG assay as an LDT, per CLSI guidelines and standards set by CAP, CLIA, and the NY state [23]. The EG multiplex assay (utilizing the current gene amplification method) has an analytical sensitivity of 89%, analytical specificity of 100%, and analytical accuracy of 96% when compared to the singleplex assay (older amplification method). Additionally, the assay is linear (*VIM*, R^2^ = 0.998 and *CCNA1* R^2^ = 0.918) and has a limit of detection as low as 1 methylated cell in the background of 400 unmethylated cells, as measured using contrived cell line mixtures. The assay displayed 100% inter- and intra-assay precision in all tested samples (both contrived and clinical samples). The samples collected with EC and preserved in room temperature media displayed the expected results for up to 21 days of storage. The assay has shown concordant results in DNA input as low as 50 ng. The EG assay met all requirements as per the standards of CAP, CLIA, and the NY state to be approved as a laboratory-developed test (LDT). 

The binary nature of test results may be viewed as a potential limitation of the EG assay given the possible uncertainty around the accuracy of results, where the methylation percentage falls close to the cut-off. We performed a quantification of samples in a well-defined grey zone to assess the appropriateness of EG as a binary assay. Only 1.7% of samples in a real-world cohort were in the grey zone for both the *VIM* and *CCNA1* genes, confirming that providing a binary EG result is suitable for nearly all samples. A comparison of EG binary results with EGD (i.e., clinical) results in samples where the underlying methylation % fell in the grey zone for either one or both genes showed that 57% of EG results were clinically accurate in this zone while 40% were false positives and only 3% of EG results were false negative. This demonstrates the assay methylation cutoff favors sensitivity over specificity. These findings are supported by the negative predictive value of 98.6% and positive predictive value of ~30% observed for EG in two clinical studies conducted within the intended use population (Table 1) [17,19]. EG was designed as a non-endoscopic tool for the initial ‘screening’ or detection of BE/EAC in patients with multiple risk factors, and a high NPV provides confidence that patients who test negative are likely to be true negative for the disease. However, since patients targeted for EG testing are those who are unlikely to have ever undergone traditional endoscopic evaluation for BE/EAC due to a low penetrance of upper endoscopy for screening among high-risk patients, the clinical impact of any potential false negatives (however rare) is further minimized [14,15]. Additionally, patients receiving false positive results by EG are not exposed to any additional risk, since they are merely referred to the gold standard screening test, EGD. As such, we have determined that EG binary results are appropriate for use of this assay as a triage test. Moreover, assigning an “indeterminate” result for patients in the grey zone would not provide the physicians with an actionable result; therefore, it would diminish the clinical utility of EG. 

The limitations of the analytical studies that we performed include that the EG assay is performed at a single laboratory and therefore the accuracy of the multiplex assay was assessed against the validated singleplex assay as a gold standard instead of a multiplex assay performed at an outside laboratory. A second limitation was that *VIM* and *CCNA1* methylation were more variable closer to the cutoff as evidenced by a higher %CV in low-positive samples. However, as the inter and intra-assay precision for EG binary results were 100% for all samples, including samples near the cutoff, a higher %CV does not impact the EG results. This is because (a) the results are based on both the *VIM* and *CCNA1* genes and (b) the results are based on triplicate data points. Finally, although our studies showed concordant EG results for DNA input as low as 50 ng, the minimum DNA input requirement of the commercial laboratory for conducting the assay is 100 ng. This is because the supporting clinical validation studies were performed using a minimum of 100 ng DNA input. Additional clinical studies would be required to eventually allow for a reduction in the minimum DNA assay requirement in the future. 

In summary, the analytical validation studies that we conducted have shown the high analytical accuracy, repeatability, and reproducibility of the EsoGuard assay. These results are further supported by the consistent clinical performance shown by the assay in three recently published independent clinical validation studies [17,18,19]. Therefore, we can conclude that EG is a robust DNA biomarker assay for the non-endoscopic detection of BE/EAC.

## Figures and Tables

**Table 1:** Summary of EsoGuard/EsoCheck performance in clinical validation studies.

	EG/EC Testing Performed in Academic Lab	EG/EC Testing Performed in Commercial Lab		
Study Name	STM (Pivotal) study [16]	BETRNet Study [18]	Cleveland VA Study [17]	Lucid BE1 Study [19]
Study Design	Case–control study	Case–control study	Single-arm study in intended use population	Single-arm study in intended use population
Evaluable Subjects (n)	Total: 86 (36 controls and 50 cases)	Total: 243 (155 controls and 88 cases)	Total: 111	Total: 93
Disease Distribution	42 BE and 8 EAC cases	70 BE and 18 EAC cases	12 BE and 2 EAC cases	8 BE cases
BE and EAC Sensitivity (95%CI)	88.0% (N/A)	85% (78–93)	92.9% (66.1–99.8)	87.5% (47.4–99.7)
Specificity (95%CI)	91.7% (N/A)	85% (79–90)	72.2% (62.1–80.8)	81.2% (71.2–88.8)
PPV (95%CI)	N/A (N/A)	38.6% (N/A) *	32.5% (18.6–49.1)	30.4% (13.2–52.9)
NPV (95%CI)	N/A (N/A)	98.1% (N/A) *	98.6% (92.4–100)	98.6% (92.3–99.96)

* PPV/NPV in case–control study was calculated using estimated disease prevalence of 10%. N/A = not available.

**Table 2:** (**A**) Methylation percentage for all 10 CpG sites measured on the *VIM* gene using the EsoGuard^®^ assay. (**B**) Methylation percentage for all 21 CpG sites measured on the *CCNA1* gene using the EsoGuard^®^ assay. (**C**) Accuracy of the EsoGuard^®^ assay pipeline in comparison to Sanger sequencing across all 10 *VIM* and 21 *CCNA1* CpG sites.

(A)								
Methylation Site #	H-1975 (0% Methylation)				SK-GT-4 (100% Methylation)			
	Rep1	Rep2	Rep3	%CV	Rep1	Rep2	Rep3	%CV
%*VIM*-Meth at POS (17271467)-CpG1	0.2%	0.2%	0.3%	24.7%	96.7%	96.8%	97.2%	0.3%
%*VIM*-Meth at POS (17271470)-CpG2	0.5%	0.3%	0.3%	31.5%	96.2%	96.4%	97.2%	0.5%
%*VIM*-Meth at POS (17271475)-CpG3	0.5%	0.9%	0.4%	44.1%	97.3%	96.4%	96.7%	0.5%
%*VIM*-Meth at POS (17271482)-CpG4	0.2%	0.4%	0.4%	34.6%	97.3%	97.4%	96.8%	0.3%
%*VIM*-Meth at POS (17271484)-CpG5	0.3%	0.5%	0.3%	31.5%	95.6%	97.3%	97.0%	0.9%
%*VIM*-Meth at POS (17271489)-CpG6	0.2%	0.3%	0.3%	21.7%	97.0%	95.9%	96.6%	0.6%
%*VIM*-Meth at POS (17271493)-CpG7	0.2%	0.3%	0.3%	21.7%	96.5%	96.7%	97.3%	0.4%
%*VIM*-Meth at POS (17271504)-CpG8	0.5%	0.5%	0.4%	12.4%	97.0%	96.8%	97.2%	0.2%
%*VIM*-Meth at POS (17271512)-CpG9	0.3%	0.5%	0.4%	25.0%	97.2%	96.1%	97.2%	0.7%
%*VIM*-Meth at POS (17271520)-CpG10	0.3%	0.5%	0.4%	25.0%	97.2%	97.0%	97.2%	0.1%
**(B)**								
**Methylation Site #**	**H-1975 (0% Methylation)**				**SK-GT-4 (100% Methylation)**			
	**Rep1**	**Rep2**	**Rep3**	**%CV**	**Rep1**	**Rep2**	**Rep3**	**%CV**
%*CCNA1*-Meth at POS (37005878)-CpG1	0.5%	0.7%	0.8%	22.9%	96.3%	96.1%	95.6%	0.4%
%*CCNA1*-Meth at POS (37005881)-CpG2	0.5%	0.8%	0.5%	28.9%	96.5%	98.1%	97.1%	0.8%
%*CCNA1*-Meth at POS (37005883)-CpG3	0.8%	0.7%	0.7%	7.9%	95.9%	95.9%	95.5%	0.2%
%*CCNA1*-Meth at POS (37005892)-CpG4	0.3%	0.4%	0.5%	25.0%	96.6%	95.2%	95.9%	0.7%
%*CCNA1*-Meth at POS (37005899)-CpG5	0.9%	0.7%	0.4%	37.7%	97.3%	96.1%	97.0%	0.6%
%*CCNA1*-Meth at POS (37005909)-CpG6	1.1%	0.4%	0.6%	51.5%	96.7%	95.4%	95.8%	0.7%
%*CCNA1*-Meth at POS (37005924)-CpG7	0.5%	0.5%	0.7%	20.4%	90.2%	91.4%	87.0%	2.5%
%*CCNA1*-Meth at POS (37005929)-CpG8	0.5%	0.9%	1.2%	40.5%	92.0%	92.2%	91.0%	0.7%
%*CCNA1*-Meth at POS (37005934)-CpG9	0.8%	0.6%	0.6%	17.3%	95.8%	94.3%	95.1%	0.8%
%*CCNA1*-Meth at POS (37005939)-CpG10	0.3%	0.8%	0.8%	45.6%	95.9%	95.5%	95.0%	0.5%
%*CCNA1*-Meth at POS (37005941)-CpG11	0.3%	0.8%	0.6%	44.4%	97.0%	96.9%	97.3%	0.2%
%*CCNA1*-Meth at POS (37005947)-CpG12	0.3%	0.2%	0.4%	33.3%	92.1%	92.4%	93.1%	0.6%
%*CCNA1*-Meth at POS (37005955)-CpG13	0.5%	0.4%	0.7%	28.6%	93.6%	91.8%	93.9%	1.2%
%*CCNA1*-Meth at POS (37005966)-CpG14	1.5%	0.9%	0.9%	31.5%	96.2%	95.5%	95.2%	0.5%
%*CCNA1*-Meth at POS (37005969)-CpG15	0.9%	0.8%	1.5%	35.5%	96.2%	94.9%	95.0%	0.8%
%*CCNA1*-Meth at POS (37005978)-CpG16	1.7%	0.9%	1.0%	36.3%	97.4%	96.5%	97.4%	0.5%
%*CCNA1*-Meth at POS (37005986)-CpG17	0.8%	1.1%	1.0%	15.8%	95.0%	94.8%	95.5%	0.4%
%*CCNA1*-Meth at POS (37005995)-CpG18	0.7%	1.5%	0.5%	58.8%	96.6%	96.0%	95.7%	0.5%
%*CCNA1*-Meth at POS (37005997)-CpG19	0.6%	0.8%	0.5%	24.1%	97.6%	96.7%	97.2%	0.5%
%*CCNA1*-Meth at POS (37006000)-CpG20	0.7%	0.4%	1.0%	42.86%	97.5%	97.0%	97.2%	0.26%
%*CCNA1*-Meth at POS (37006008)-CpG21	0.6%	0.2%	0.5%	48.0%	96.8%	97.3%	96.0%	0.7%
**(C)**								
	***VIM***				***CCNA1***			
	**Sanger Sequencing**		**Average EsoGuard Assay Computed Methylation % Across All 10 CpG Sites**		**Sanger Sequencing**		**Average EsoGuard Assay Computed Methylation % Across All 21 CpG Sites**	
H-1975	0%		0%		0%		0%	
SK-TG-4	100%		99.5%		100%		99.4%	

**Table 3:** (**A**) Analytical accuracy of the EsoGuard^®^ assay in comparison to CWRU reference lab: balloon samples. (**B**) Analytical accuracy of EsoGuard^®^ assay in comparison to CWRU reference lab: contrived samples. (**C**) Analytical performance of EsoGuard^®^ assay (singleplex) against CWRU reference lab.

(A)			
Analytical Accuracy (Singleplex)—EsoCheck Balloon Samples		EsoGuard^®^ Assay Performed at Reference Lab (CWRU)	
		Positive	Negative
EsoGuard assay performed at test lab (LucidDx Labs)	Positive	32	3
	Negative	1	13
**(B)**			
**Analytical Accuracy (Singleplex)—Contrived Samples**		**EsoGuard^®^ Assay Performed at Reference Lab (CWRU)**	
		**Positive**	**Negative**
EsoGuard assay performed at test lab (LucidDx Labs)	Positive	33	0
	Negative	0	7
**(C)**			
	**EsoCheck Balloon Samples** **(*n* = 49)**	**Contrived Samples** **(*n* = 40)**	**Overall** **(*n* = 89)**
Analytical Sensitivity	96.9%	100%	98.5%
Analytical Specificity	81.3%	100%	87.0%
Analytical Accuracy	91.8%	100%	95.5%

**Table 4:** Analytical accuracy of the multiplex EsoGuard^®^ assay in comparison to singleplex.

Analytical Accuracy (Multiplex)—EsoCheck Balloon Samples		EsoGuard Assay (Singleplex)		Analytical Accuracy
		Positive	Negative	
EsoGuard Assay (Multiplex)	Positive	24	0	96.1%
	Negative	3	50	
Analytical Sensitivity and Specificity		88.9%	100%	

**Table 5:** Intra-assay precision.

Intra-Assay	Genes	Average (Methylation%)	Standard Deviation	%CV	EG Assay Concordance
Contrived—10% (Medium positive)	*VIM*	29.2	0.4	1%	C (3/3)
	*CCNA1*	52.6	4.8	9%	
Contrived—1% (Low-positive)	*VIM*	7.9	2.5	32%	C (3/3)
	*CCNA1*	12.4	5.3	42%	
Contrived—0.5% (Low-positive)	*VIM*	3.2	1.1	34%	C (4/4)
	*CCNA1*	5.2	3.5	67%	
Contrived—0.0% * (Negative)	*VIM*	0.0	0.0	0%	C (3/3)
	*CCNA1*	0.0	0.0	0%	
EsoCheck Balloon Positive-1	*VIM*	30.6	1.0	3%	C (3/3)
	*CCNA1*	28.9	3.2	11%	
EsoCheck Balloon Positive-2	*VIM*	27.5	1.3	5%	C (3/3)
	*CCNA1*	28.7	7.7	27%	
EsoCheck Balloon Positive-3	*VIM*	3.0	0.8	28%	C (3/3)
	*CCNA1*	1.1	0.4	39%	
EsoCheck Balloon Negative-1 *	*VIM*	0.0	0.0	0%	C (3/3)
	*CCNA1*	0.0	0.0	0%	
EsoCheck Balloon Negative-2 *	*VIM*	0.0	0.0	0%	C (3/3)
	*CCNA1*	0.0	0.0	0%	
EsoCheck Balloon Negative-3 *	*VIM*	0.0	0.1	0%	C (3/3)
	*CCNA1*	0.0	0.0	0%	

* %CV could not be calculated due to division by 0 and has been converted to 0% manually.

**Table 6:** Inter-assay precision.

Inter-Assay	Genes	Average (Methylation%)	Standard Deviation	%CV	Assay Concordance
Contrived—10% (Medium positive)	*VIM*	36.9	9.8	27%	C (3/3)
	*CCNA1*	50.5	3.1	6%	
Contrived—1% (Low positive)	*VIM*	8.8	1.1	13%	C (3/3)
	*CCNA1*	11.3	1.7	16%	
Contrived—0.5% (Low positive)	*VIM*	4.8	1.6	33%	C (3/3)
	*CCNA1*	7.8	3.0	38%	
Contrived—0.0% * (Negative)	*VIM*	0.0	0.0	0%	C (3/3)
	*CCNA1*	0.0	0.0	0%	
EsoCheck Balloon Positive-1	*VIM*	30.9	1.5	5%	C (3/3)
	*CCNA1*	23.9	2.3	9%	
EsoCheck Balloon Positive-2	*VIM*	22.9	3.0	13%	C (3/3)
	*CCNA1*	19.7	0.4	2%	
EsoCheck Balloon Positive-3	*VIM*	2.4	0.5	21%	C (3/3)
	*CCNA1*	1.5	0.6	45%	
EsoCheck Balloon Negative-1 *	*VIM*	0.0	0.0	0%	C (3/3)
	*CCNA1*	0.0	0.0	0%	
EsoCheck Balloon Negative-2 *	*VIM*	0.0	0.0	0%	C (3/3)
	*CCNA1*	0.0	0.0	0%	
EsoCheck Balloon Negative-3 *	*VIM*	0.1	0.1	0%	C (3/3)
	*CCNA1*	0.0	0.0	0%	

* %CV could not be calculated due to division by 0 and has been converted to 0% manually.

**Table 7:** Limit of detection (LOD).

	*n*	Average *VIM*	Average *CCNA1*	# of *VIM* Positive	# *CCNA1* Positive	EsoGuard^®^ Positive
1P-Spike-in	24	9.5	10.8	24	24	24 (100%)
0.5P-Spike-in	27	4.8	7.4	26	20	27 (100%)
0.25P-Spike-in	24	3.0	5.6	21	21	24 (100%)
0.13P-Spike-in	20	1.6	2.2	13	12	18 (90%)

**Table 8:** Reference range.

	EGD Result	
	Positive	Negative
EsoGuard Assay Positive	0	13
EsoGuard Assay Negative	0	69
Specificity	84.1%	

## Data Availability

The data presented in this study are available on request from the corresponding author due to proprietary information.

## Supplementary Materials

The following supporting information can be downloaded at: https://www.mdpi.com/article/10.3390/diagnostics14161784/s1, Figure S1: Sanger Sequencing data showing all 31 CpG sites are methylated in SK-GT-4 cell line (Sbjct 1) and not methylated in H1975 cell line (Query 1); Figure S2: EsoGuard bioinformatics pipeline accuracy (v 2.0) in comparison to previous version of the pipeline; Figure S3: Methylation percentages of NextSeq 1000 platform against MiSeq platform; Table S1. Analytical accuracy of multiplex EsoGuard® (EG) assay in comparison to singleplex; Table S2. Sample Stability in Preservative media. 1% contrived specimen (1 P) and 0% contrived specimen (0P) were tested at Day 0 (D0), Day 2(D2), Day 14 (D14) and Day 21 (D21); Table S3. Sample Stability in Preservative media at different day 1 and day 2 temperatures: results on day14.
