# Supplementary material for: Analytical Validation of a DNA Methylation Biomarker Test for the Diagnosis of Barrett’s Esophagus and Esophageal Adenocarcinoma from Samples Collected Using EsoCheck®, a Non-Endoscopic Esophageal Cell Collection Device
Source: Diagnostics (Basel). 2024 Aug 15;14(16):1784. doi: 10.3390/diagnostics14161784 (PMC11354049; doi:10.3390/diagnostics14161784)
Supplement: Supplementary file 1 [file diagnostics-14-01784-s001.zip › diagnostics-3088910-supplementary.pdf]

Supplementary Materials:

Supplementary Figures:

Figure S1. Sanger Sequencing data showing all 31 CpG sites are methylated in SK-GT-4 cell line (Sbjct 1) and not methylated in H1975 cell line (Query 1).

H1975 (0%) vs. SK-GT-4 (100%)

```
Query  1      CACAG-TTGTTTAGGTTGTAGGTGCNGGGTGGATGTAGTTATGTAGTTTTGGTTGGAGT  58
              || || ||||||||||||||||||||||||||| ||||||| ||||||| |||||||||
Sbjct  1     GCATAGGTTGTTTAGGTTGTAGGTGCNGGGTGGACGTAGTTACGTAGTTTCGGTTGGAGT  60

Query  59    TTGNTTGNTTTGTGGTGTTTGGGTTGTTGAATATTT   94
             | | | | || | |||||| |||| || ||||||||
Sbjct  61    TCGGTCGGTTCGCGGTGTTCGGGTCGTCGAATATTTT  97
```

H1975 (0%) vs. SK-GT-4 (100%)

```
Query  1     CG---------ATTGTATTTGGGGTAGTTTTGTTGTGTTTTAGTTGTTTTTTGGTAGGAA  51
              |         ||||||||||||||||||| || | |||||||| |||||| ||||||||
Sbjct  1     TGGCATNNGNGATTGTATTTGGGGTAGTTTCGTCGCGTTTTAGTCGTTTTTCGGTAGGAA  60

Query  52    GTGTAGGTGTGTGAGTTGATTTGGAGTGAGTTGTGTTTTTGGGTTAGTGTGGGTAGGGTG  111
             | |||||||||||||| |||| | || |||| | ||||| ||||||| |||||||||| |
Sbjct  61    GCGTAGGTGTGTGAGTCGATTCGNAGCGAGTCGCGTTTTCGGGTTAGCGTGGGTAGGGCG  120

Query  112   TTGTAGTTTGTGTAGTTTTGAGGATTTTGTGTTGTTTTTTTGAGTTAGGGTTTTTAGGAG  171
             | |||||||| ||||||| |||||||| | || ||||||| ||||||||||||||||||
Sbjct  121   TCGTAGTTTGCGTAGTTTCGAGGATTTCGCGTCGTTTTTTCGAGTTAGGGTTTTTAGGA   179

Query  172   CGGGTGTGTATA  183
```

Figure S2. EsoGuard bioinformatics pipeline accuracy (v 2.0) in comparison to previous version of the pipeline.

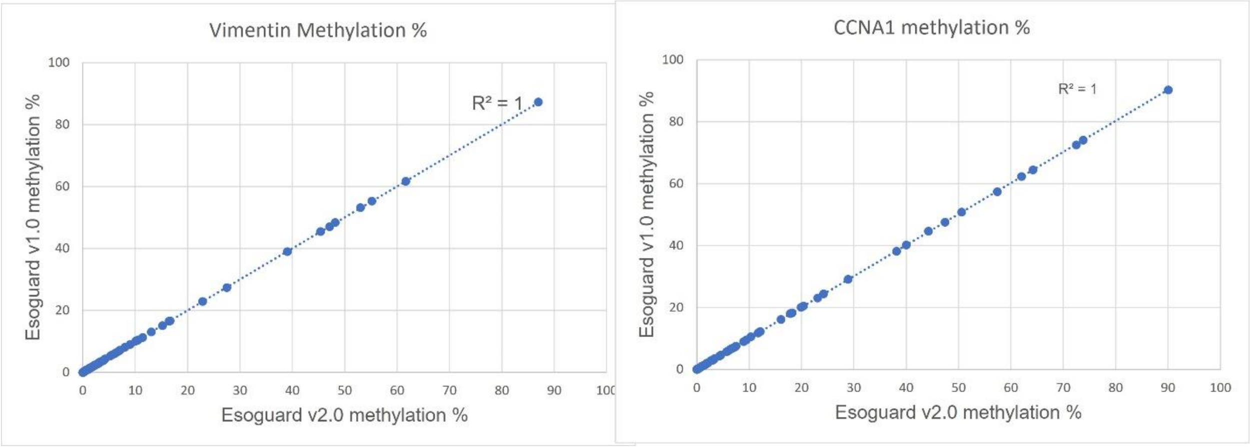

Figure S3. Methylation percentages of NextSeq 1000 platform against MiSeq platform.

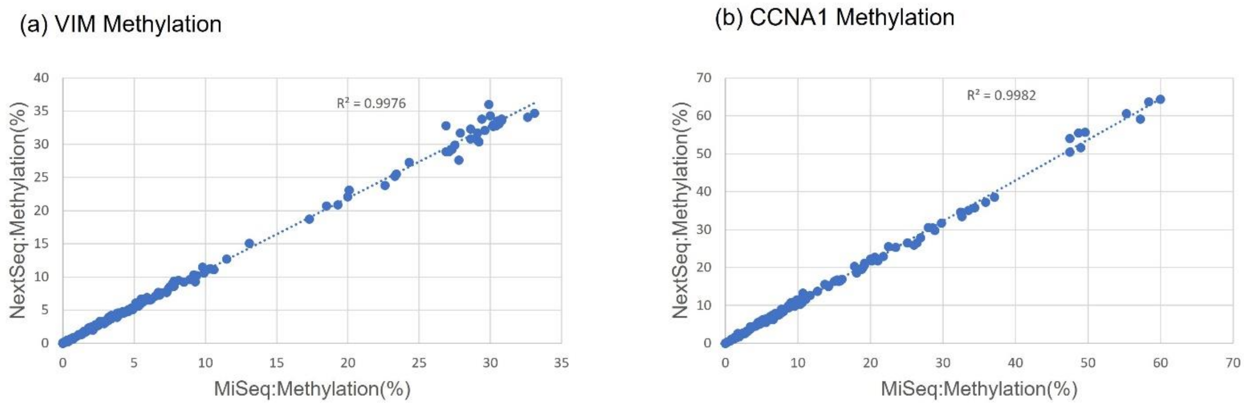

Supplementary Tables:

Table S1. Analytical accuracy of multiplex EsoGuard® (EG) assay in comparison to singleplex.

Patient No.	VIM Methylation Percent				CCNA1 Methylation Percent				EG Binary Result (Singleplex)	EG Binary Result (Multiplex)	Concor dance (Y/N)
	EG (Single-plex)	EG (Multi-plex) Rep1	EG (Multi-plex) Rep2	EG (Multi-plex) Rep3	EG (Single-plex)	EG (Multi-plex) Rep1	EG (Multi-plex) Rep2	EG (Multi-plex) Rep3			
EC-001	8.3	6.3	4.6	7.1	4.8	5.8	6.1	1.9	Positive	Positive	Y
EC-002	3.2	4.1	2.6	5.1	5.9	9	6.8	6.2	Positive	Positive	Y
EC-003	0	0	0	0	0	0	0	0	Negative	Negative	Y
EC-004	0.2	0.2	0.2	0	0	0	0	0	Negative	Negative	Y
EC-005	0	0	0	0	0	0	0	0	Negative	Negative	Y
EC-006	0.1	0	0	0	0	0	0	0	Negative	Negative	Y
EC-007	0	0	0	0	0	0	0	0	Negative	Negative	Y
EC-008	0	0	0	0	0	0	0	0	Negative	Negative	Y
EC-009	0	0	0.1	0	0.1	0	0	0	Negative	Negative	Y
EC-010	0.4	0.3	0.5	0.4	0	0	0	0	Negative	Negative	Y
EC-011	0.3	0	0	0.4	0.1	0	0	0.9	Negative	Negative	Y
EC-012	4.1	4.8	5.7	3.6	9.9	6.9	3.7	5.2	Positive	Positive	Y
EC-013	20.4	18.2	17.7	20.3	19.9	35.6	23	26.7	Positive	Positive	Y
EC-014	2	3.7	4.2	4.8	0.4	3	1.2	1.8	Positive	Positive	Y
EC-015	5.4	7.1	4.3	5.6	5.1	3.6	5.8	9.5	Positive	Positive	Y
EC-016	0	0	0	0	0	0	0	0	Negative	Negative	Y
EC-017	0.1	0.4	0	0	0	0	0	0	Negative	Negative	Y
EC-018	0.1	0.4	0.5	0.1	0	0	0	0	Negative	Negative	Y
EC-019	0	0	0	0	0	0	0.5	0	Negative	Negative	Y
EC-020	9.4	4.5	5.4	4.1	0.5	3.9	1.1	1.7	Positive	Positive	Y
EC-021	0	0	0	0	0	0	0	0	Negative	Negative	Y
EC-022	0.4	0.6	0.7	0.6	0	0	0.1	0	Negative	Negative	Y
EC-023	24.2	15.1	16.8	18.5	27.9	38.8	26.7	35.4	Positive	Positive	Y
EC-024	1.2	0.6	0.1	1.0	0	0	0	0	Positive	Negative	N
EC-025	0	0.2	0	0	0	0	0.4	0	Negative	Negative	Y
EC-026	0.1	0	0	0	0	0	0	0	Negative	Negative	Y
EC-027	0	0	0	0	0	0	0	0	Negative	Negative	Y
EC-028	6.7	7.9	11.2	9.4	9.1	10.8	12.5	14.9	Positive	Positive	Y
EC-029	0	0	0	0	0	0	0	0	Negative	Negative	Y
EC-030	6.3	8.1	6.2	6.7	8.8	7.6	8.2	7.3	Positive	Positive	Y
EC-031	5	7.4	7.6	7.7	3.3	3.2	4.1	6.9	Positive	Positive	Y
EC-032	0.6	0.7	0	0.3	0	0	0	0	Negative	Negative	Y
EC-033	0	0	0	0	0	0	0	0	Negative	Negative	Y
EC-034	0.2	0	0	0	0	0	0	0	Negative	Negative	Y
EC-035	0.3	0.1	0.1	0	0	0	0	0	Negative	Negative	Y

EC-036	0	0.1	0	0	0	0	0	0	Negative	Negative	Y
EC-037	0.1	0	0	0	0	0	0	0	Negative	Negative	Y
EC-038	13.2	12.4	12	10.5	14.3	25.6	22.3	21.1	Positive	Positive	Y
EC-039	5.7	4.7	3.2	2.9	1.7	6.7	9.4	10.4	Positive	Positive	Y
EC-040	8.9	5.9	5.4	5.3	7	7.1	12.9	9.8	Positive	Positive	Y
EC-041	8.1	4.1	3.5	4.3	5.2	5.6	7.4	4.1	Positive	Positive	Y
EC-042	0	0	0	0	0	0	0	0	Negative	Negative	Y
EC-043	0	0	0	0	0	0	0	0	Negative	Negative	Y
EC-044	0.2	0.2	0.2	0.2	0	0	0	0	Negative	Negative	Y
EC-045	0.6	0.4	0.3	0.5	0	0	0	0	Negative	Negative	Y
EC-046	22	18.7	17.8	20.6	25.1	13.8	14.5	14	Positive	Positive	Y
EC-047	16.8	15.4	12.6	21.6	16.9	12.8	11.1	12.9	Positive	Positive	Y
EC-048	8.7	1.6	0.6	1.1	2.5	3.5	2	1.4	Positive	Positive	Y
EC-049	4.9	7.7	4.6	4.6	3.7	4.3	3.3	2.2	Positive	Positive	Y
EC-050	0.6	0.2	0	0.3	0	0	0	0	Negative	Negative	Y
EC-051	0.1	0	0.1	0.1	0	0	0	0	Negative	Negative	Y
EC-052	0	0.2	0.1	0.1	0	0	0	0	Negative	Negative	Y
EC-053	0.4	0.1	0.1	0.3	0	0	0	0	Negative	Negative	Y
EC-054	1.1	0	0	0	0	0	0	0	Positive	Negative	N
EC-055	0.1	0	0	0.2	0	0	0	0	Negative	Negative	Y
EC-056	0	0	0.1	0.1	0	0	0	0	Negative	Negative	Y
EC-057	0	0	0	0	0	0	0	0	Negative	Negative	Y
EC-058	0	0	0	0	0	0	0	0	Negative	Negative	Y
EC-059	0.6	0	0.1	0.6	0	0	0	0.1	Negative	Negative	Y
EC-060	0.1	0.1	0	0	0	0	0	0	Negative	Negative	Y
EC-061	7.9	3.2	2.5	2.4	5.7	2.8	2.3	1.2	Positive	Positive	Y
EC-062	2.9	1.3	1.1	1	6.1	8.6	8.5	8.8	Positive	Positive	Y
EC-063	4.7	4	5.1	6.1	2.3	1.4	2.4	2.1	Positive	Positive	Y
EC-064	1.1	0.3	0.5	0.3	0	0	0	0	Positive	Negative	N
EC-065	16.7	11	11.1	12.2	32.1	19.8	22.2	21.5	Positive	Positive	Y
EC-066	0.1	0	na	0.3	0	0	na	0	Negative	Negative	Y
EC-067	0	0	0	0	0	0	0	0	Negative	Negative	Y
EC-068	0	0.4	0.2	1	0	0	0	0	Negative	Negative	Y
EC-069	0.9	0.5	0.5	0.4	0.1	0	0.1	0	Negative	Negative	Y
EC-070	0	0	0	0	0	0	0	0	Negative	Negative	Y
EC-071	0.2	0.1	0	0	0	0	0	0	Negative	Negative	Y
EC-072	8.8	6.8	6.7	5.2	8.7	5.7	7.3	5.9	Positive	Positive	Y
EC-073	0	0	0	0	0	0	0	0	Negative	Negative	Y
EC-074	0	0	0	0	0	0	0	0	Negative	Negative	Y
EC-075	0	0	0	0	0	0	0	0	Negative	Negative	Y
EC-076	0	0	0	0	0	0	0	0	Negative	Negative	Y
EC-077	0.5	0.1	0.2	0.1	0	0	0	0	Negative	Negative	Y

*na=non-diagnostic result due to QC failure

Table S2. Sample Stability in Preservative media. 1% contrived specimen (1 P) and 0% contrived specimen (0P) were tested at Day 0 (D0), Day 2(D2), Day 14 (D14) and Day 21 (D21).

Sample ID	Vim % Rep1	Vim % Rep2	Vim % Rep3	CCNA1 % Rep1	CCNA1 % Rep2	CCNA1 % Rep3	Status	Concordance (Y/N)
D0-0P-1	0	0	0	0	0	0	Negative	Y
D0-0P-2	0	0	0	0	0	0	Negative	Y
D0-0P-3	0	0	0	0	0	0	Negative	Y
D2-0P-1	0	0	0	0	0	0	Negative	Y
D2-0P-2	0	0	0	0	0	0	Negative	Y
D2-0P-3	0	0	0	0	0	0	Negative	Y
D7-0P-1	0	0	0	0	0	0	Negative	Y
D7-0P-2	0	0	0	0	0	0	Negative	Y
D7-0P-3	0	0	0	0	0	0	Negative	Y
D14-0P-1	0	0	0	0	0	0	Negative	Y
D14-0P-2	0	0	0	0	0	0	Negative	Y
D14-0P-3	0	0	0	0	0	0	Negative	Y
D21-0P-1	0	0	0	0	0	0	Negative	Y
D21-0P-2	0	0	0	0	0	0	Negative	Y
D21-0P-3	0	0	0	0	0	0	Negative	Y
D0-1P-1	8.9	9.3	9.5	13.2	18.3	5.4	Positive	Y
D0-1P-2	10	9.7	10	13.9	12.1	13.3	Positive	Y
D0-1P-3	8.9	5.4	9.6	19.9	15.1	7.7	Positive	Y
D2-1P-1	15.5	9.3	14.8	22.6	17.5	23.8	Positive	Y
D2-1P-2	10.4	8.2	10.1	16.2	20.4	21.3	Positive	Y
D2-1P-3	12	9.9	11.9	20.3	20.5	23.1	Positive	Y
D7-1P-1	8.5	7.4	8	29.2	43.8	23.6	Positive	Y
D7-1P-2	10.2	9	10.9	30.9	27.6	37.2	Positive	Y
D7-1P-3	17.4	12.9	16.5	27.3	24.3	19.2	Positive	Y
D14-1P-1	13.9	15.8	13.4	28.9	27.4	17.3	Positive	Y
D14-1P-2	16.9	14.2	13	25.4	24.3	20.2	Positive	Y
D14-1P-3	13.8	16	15	27.1	25.3	31.8	Positive	Y
D21-1P-1	9.4	8.6	14.4	12.3	29.6	18.5	Positive	Y
D21-1P-2	14.2	14.8	13.6	15.8	18.3	20.9	Positive	Y
D21-1P-3	11.1	13	17.8	27.1	20.2	23.4	Positive	Y

Table S3. Sample Stability in Preservative media at different day 1 and day 2 temperatures: results on day14.

Sample ID	Vim % Rep1	Vim % Rep2	Vim % Rep3	CCNA1 % Rep1	CCNA1 % Rep2	CCNA1 % Rep3	Status	Concordance (Y/N)
D14-0P-50C-1	0	0	0	0	0	0	Negative	Y
D14-0P-50C-2	0	0	0	0	0	0	Negative	Y
D14-0P-50C-3	0	0	0	0	0	0	Negative	Y
D14-0P-37C-1	0	0	0	0	0	0	Negative	Y
D14-0P-37C-2	0	0	0	0	0	0	Negative	Y
D14-0P-37C-3	0	0	0	0	0	0	Negative	Y
D14-0P-RT-1	0	0	0	0	0	0	Negative	Y
D14-0P-RT-2	0	0	0	0	0	0	Negative	Y
D14-0P-RT-3	0	0	0	0	0	0	Negative	Y
D14-0P-4C-1	0	0	0	0	0	0	Negative	Y
D14-0P-4C-2	0	0	0	0	0	0	Negative	Y
D14-0P-4C-3	0	0	0	0	0	0	Negative	Y
D14-0P-Neg20C-1	0	0	0	0	0	0	Negative	Y
D14-0P-Neg20C-2	0	0	0	0	0	0	Negative	Y
D14-0P-Neg20C-3	0	0	0	0	0	0	Negative	Y
D14-1P-50C-1	11.2	8.9	9.7	23.2	15.9	15.8	Positive	Y
D14-1P-50C-2	10.8	9.8	10.5	25.8	26.2	24.4	Positive	Y
D14-1P-50C-3	6.8	5.6	6.1	13.8	13.6	11.1	Positive	Y
D14-1P-37C-1	14.6	11.8	14.5	22.5	15.4	23.4	Positive	Y
D14-1P-37C-2	18.5	15.1	18.3	29	18.3	22	Positive	Y
D14-1P-37C-3	9.5	8.5	9.5	30.8	33.5	22.9	Positive	Y
D14-1PRT-1	13.9	15.8	13.4	28.9	27.4	17.3	Positive	Y
D14-1P-RT-2	16.9	14.2	13	25.4	24.3	20.2	Positive	Y
D14-1P-RT-3	13.8	16	15	27.1	25.3	31.8	Positive	Y
D14-1P-4C-1	17.6	15.1	13.1	26.3	25.5	24.8	Positive	Y
D14-1P-4C-2	21	17.3	16.7	49.6	47.6	50.1	Positive	Y
D14-1P-4C-3	17.6	14	16.1	16.5	19.9	14.4	Positive	Y
D14-1P-Neg20C-1	16	19.2	14.7	17.1	21.8	14.4	Positive	Y
D14-1P-Neg20C-2	12.4	8.3	11.3	36.1	29.4	22.6	Positive	Y
D14-1P-Neg20C-3	12.8	8.6	8.9	22.9	24.3	27.5	Positive	Y
